# Differential expression of Cyclin D1 in keratin-producing odontogenic cysts

**DOI:** 10.4317/medoral.20114

**Published:** 2015-01-05

**Authors:** Beatriz Vera-Sirera, Leopoldo Forner-Navarro, Francisco Vera-Sempere

**Affiliations:** 1 PhD Student, Department of Stomatology, Valencia University, Valencia, Spain; 2 Professor of Stomatology, Department of Stomatology, Valencia University, Valencia, Spain; 3 Chair of Pathology, Department of Pathology, La Fe University Hospital, Valencia University, Valencia, Spain

## Abstract

Objetives: The aim of the present study was to analyze the expression levels of Cyclin D1 (CCD1), a nuclear protein that plays a crucial role in cell cycle progression, in a series of keratin-producing odontogenic cysts. Study Design: A total of 58 keratin-producing odontogenic cysts, diagnosed over ten years and classified according to the WHO 2005 criteria, were immunohistochemically analyzed in terms of CCD1 expression, which was quantified in the basal, suprabasal and intermediate/superficial epithelial compartments. The extent of immunostaining was measured as a proportion of total epithelial thickness. Quantified immunohistochemical data were correlated with clinicopathological features and clinical recurrence. Results: Keratin-producing odontogenic cysts were classified as 6 syndromic keratocystic odontogenic tumors (S-KCOT), 40 sporadic or non-syndromic KCOT (NS-KCOT) and 12 orthokeratinized odontogenic cysts (OOC). Immunohistochemically, CCD1 staining was evident predominantly in the parabasal region of all cystic lesions, but among-lesion differences were apparent, showing a clear expansion of parabasal compartment especially in the S-KCOT, followed to a lesser extent in the NS-KCOT, and being much more reduced in the OOC, which had the greatest average epithelial thickness. Conclusions: The differential expression of CCD1 noted in the present study suggests that dysregulation of cell cycle progression from G1 to the S phase contributes to the different aggressiveness of these lesions. However, CCD1 expression levels did not predict NS-KCOT recurrence, which is likely influenced by factors unrelated to lesion biology.

** Key words:**Keratin-producing odontogenic cyst, keratocyst, keratocystic odontogenic tumor, nevoid basal cell carcinoma syndrome, orthokeratinized odontogenic cyst, cyclin D1, immunohistochemistry.

## Introduction

Odontogenic cyst with keratinized epithelium form a heterogeneous group of cystic lesions that are often aggressive in character, with high rate of recurrence and multifocality (1). Malignant transformation is possible, albeit very rare (2) and the effective management of such lesions is sub jet of frequent discusion (3).

In the time since the last WHO classification (4), nomenclature of these lesions has been modified. It is accepted that there a true tumor, termed keratocystic odontogenic tumor (KCOT), can present as either a sporadic (non syndromic) lesion (KCOT-NS) or in a syndromic form (KCOT-S), as a component of the nevoid basal cell carcinoma syndrom (NBCCS) (1,2). Within these lesional spectrum, the WHO classification (4) also distinguishes the so-called orthokeratinized odontogenic cyst (OOC), that (unlike KCOT) does not have a syndromic association, and shows neither a tendency to recur nor multilocularity (5-8).

Genetic and molecular alterations in the SHH (sonic hedgehog) pathway (9) have been implicated in KCOT pathogenesis. Mutations in the PTCH1 gene have been noted in KCOT-S patients, and PTCH loss of heterozygosity (LOH) has also been described in those with OOC (10), suggesting that different genetic alterations are associated with various lesional subtypes. Such changes are well-correlated with the expression of various markers, including cycling D1 (CCD1) (9), a nuclear protein playing a crucial role in cell cycle progression (11) from G1 to S phase. CCD1 allows cells to progress to the DNA synthesis phase, and CCD1 is over expressed in several models of tumorigenesis (12). In the present study, we immunohistochemically analyzed CCD1 expression levels in a series of keratin-producing odontogenic cysts, classified using the WHO criteria (4), and associated the extent of protein expression with various clinicopathological subtypes within the spectrum of these lesions.

## Material and Methods

We studied 58 cases of keratin-producing odontogenic cysts, diagnosed over a period of 10 years at the Department of Pathology of La Fe University Hospital, Valencia, Spain. Histological material was retrieved from storage. Our work formed part of a project previously approved by our Ethics Committee for Biomedical Research (protocol no. 2013/0045).

We selected cases using a pathological diagnosis database (Pat Win® version 4.1.4). We performed a 10-year retrospective search employing the following search terms: “keratocyst”, “primordial cyst”, “keratocystic odontogenic tumor”, “orthokeratinized odontogenic cyst”, and “keratinized cyst”, as the terms used to describe the various lesions have changed over time.

All original histological sections were reviewed microscopically by two observers, and were reclassified using the WHO 2005 criteria (5). The lesions were diagnosed as OOC when a cystic lesion had an orthokeratinized epithelial lining, with an obvious granular layer, in the absence of any region of surface corrugation and without any tendency toward formation of cellular palisades in the basal region of the epithelium (5,6).

The clinical, radiological, and surgical data on all patients were gathered from the medical records Mizar® 2.0 platform with the aid of the viewfinder Luna® 3.0. We noted the following data: age; gender; location of the lesion (mandibular and/or maxillary); the affected zone: (anterior [between the right and left canines], premolar, or molar); clinical/radiological follow-up findings, number of recurrences; and any clinical, pathological, or genetic data suggesting a syndromic association. We used the criteria of Kimonis *et al*. (13) to diagnose NBCCS.

Sections 5 µm in thickness were cut from the original paraffin-embedded blocks and mounted on poly-L-lysine-coated glass slides prior to immunohistochemical staining. Epitope retrieval proceeded at 97ºC for 20 min in high-pH EnVision FLEX Target Retrieval solution, followed by washing for 5 min in envision FLEX Wash Buffer. CCD1 was immunostained using a rabbit monoclonal antibody (clone EP12; Dako, Glostrup, Denmark). The incubation time was 20 min and staining was visualized using the high-pH envision FLEX system. Tonsil sections served as positive staining controls and the negative controls were mock-stained test sections (the primary antibody was replaced by PBS).

CCD1 immunostaining was quantified as follows. The ratio of the depth of epithelium stained (assessed in the region of maximal expression) to the total depth of the epithelial wall (both values in µm) was calculated. The extent of immunostaining of each of the basal, parabasal and intermediate/superficial epithelial layers was evaluated as a percentage (to the nearest 10%) We recorded whether the nuclei, cytoplasm, or both, were stained. All measurements were performed at 20× magnification using a DMD108 digital microscope (Leica Microsystems, Wetzlar, Germany) featuring automatic calibration. Means ± standard deviations were calculated and the immunostaining data analyzed by reference to lesional type and (in KCOT-NS cases) the extent of recurrence.

Categorical variables are presented as numbers with percentages, and data from different groups were compared using the Pearson χ2 test or the one-sided Fisher’s exact test, as appropriate. All measurements are expressed in µm, as means ± standard deviations, and these continuous variables were compared using the Kruskal-Wallis test. We used the Poisson regression model to compare the frequencies of recurrence among groups, solving any convergence problem associated with use of the maximum likelihood algorithm via adjustment with the aid of an expectancy-maximization algorithm. All statistical analyses were performed with the aid of SPSS for Windows (version 14.0; SPSS Inc., Chicago, IL), and a *p* value ≤ 0.05 was considered to reflect statistical significance.

## Results

Upon histological review, the 58 keratin-producing odontogenic cysts were classified, using the WHO criteria, into 46 examples of KCOT and 12 of OOC. Of the 46 KCOT cases, 6 were considered to be S-KCOT because other lesions or abnormalities consistent with the presence of NBCCS were also noted.  Table 1 summarizes the epidemiological data, as well as the frequency of recurrences, based on information from the clinical and radiological follow-up.

**Table 1:**
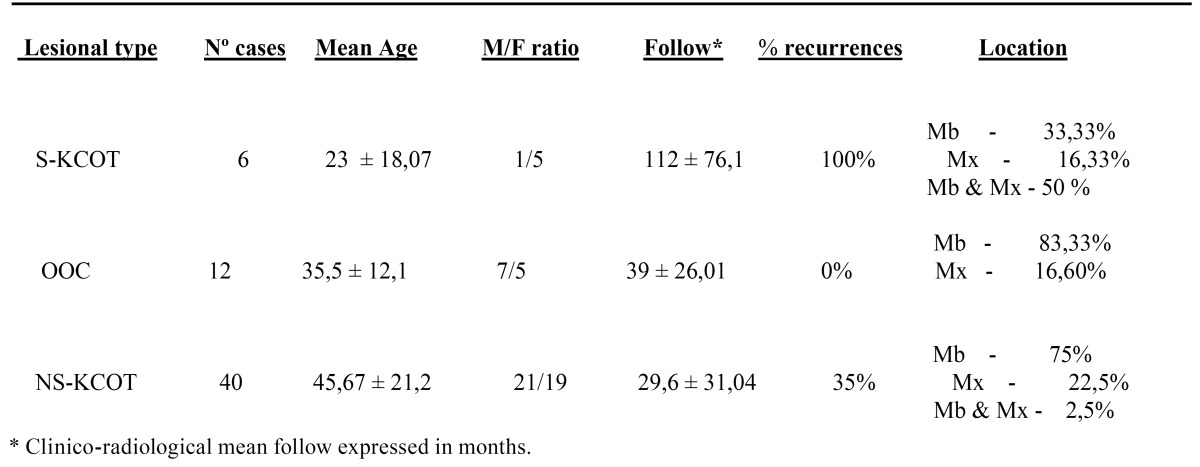
Epidemiological and follow-up data.

S-KCOT (six cases) affected five females and one male, aged 9-54 years, and the mean patient age was 23±18.07 years at the time of diagnosis. All patients experienced recurrences; the mean number of recurrences was 3±1.41 over a mean follow-up time of 112±76.1 months. Lesions occurred in the mandible and maxilla (three patients), the mandible alone (two patients), and the maxilla alone (one patient). The premolar region was the most frequently affected, followed by the molar and anterior regions.

NS-KCOT (40 cases) occurred in 21 males and 19 females (M/F ratio: 1.10) of a mean age of 45.67±21.2 years (range, 9-84 years), and 35% of patients (14 of 40) experienced recurrences (an average of 1.4±0.6 relapses) over 29.6±31.04 months of follow-up. Of all patients, 75%, 22.5%, and 2.5% had lesions in the mandible, maxilla, or both, respectively. The premolar mandibular region was most often affected and the anterior region least affected.

OOCs were noted in seven males and five females, and the mean age at diagnosis was 35.5±12.1 years (range, 19-65 years). Of all patients, 83.5% had mandibular involvement exclusively and only two had lesions in maxilla. The molar mandibular area, followed by the premolar mandibular region, was the most frequently affected site. Five of the twelve cysts (41.6%) were associated with impacted teeth and were initially clinically and radio logically misdiagnosed as dentigerous cysts. No OOC patient had multiple lesions, and no patient experienced recurrence or development of another lesion during a mean follow-up time of 39±26.1 months (range, 9-95 months).

**Figure 1:**
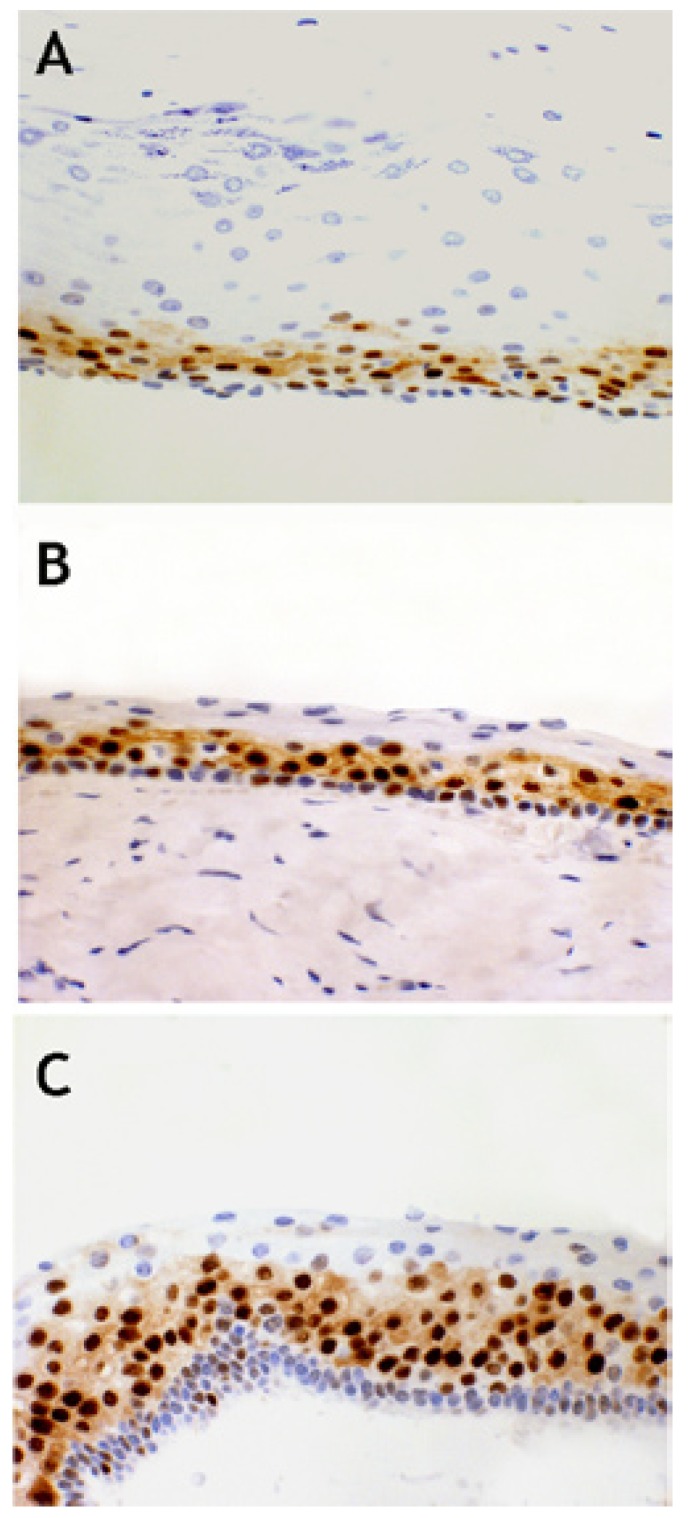
CCD1+ expression in OOC (A), NS-KCOT (B) and S-KCOT (C). CCD1 x 400.

**Figure 2:**
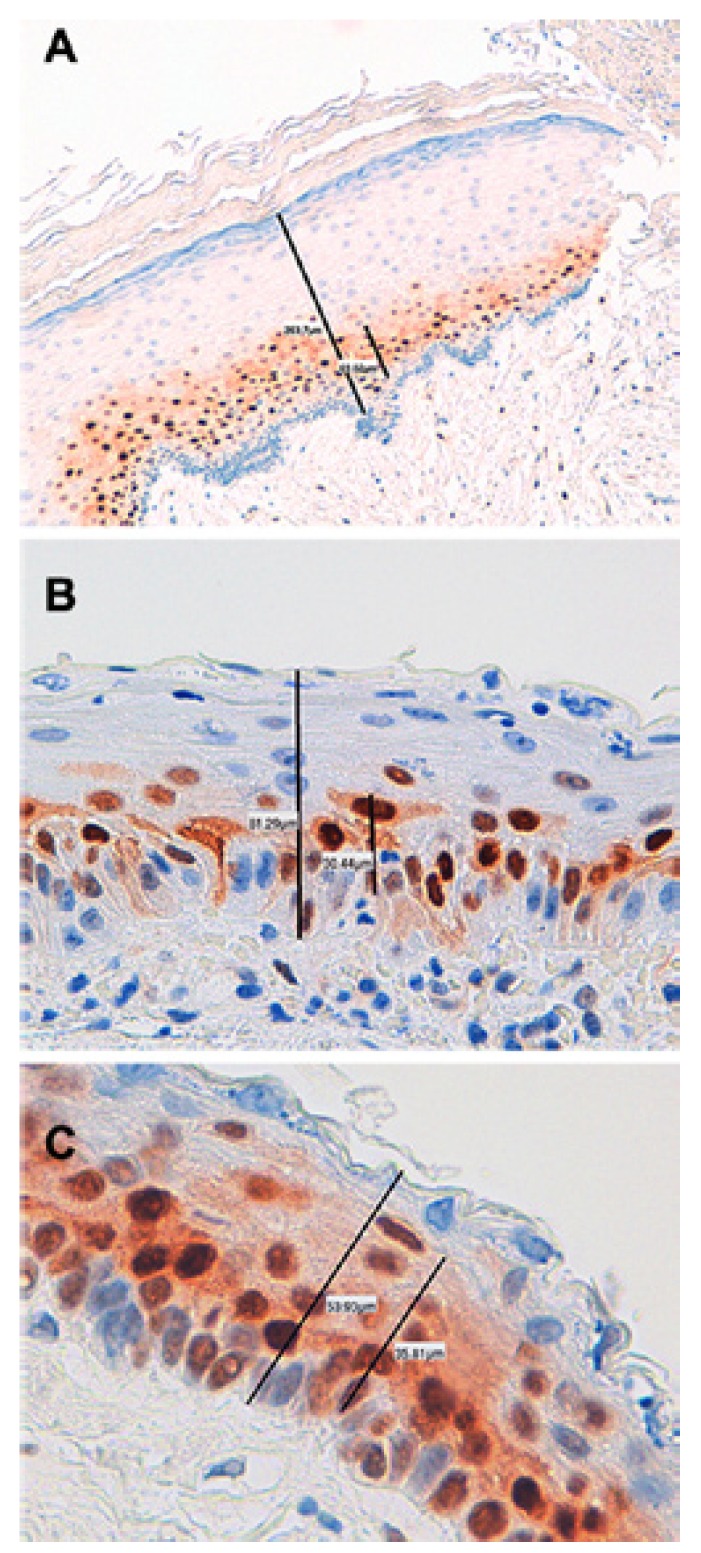
Measurements of total thickness of cystic epithelial lining and epithelial thickness with CCD1+ immunostaining performed by digital microscopy with automatic calibration, in samples of OOC (A), NS-KCOT (B) and S-KCOT (C). CCD1 250-400x.

**Figure 3:**
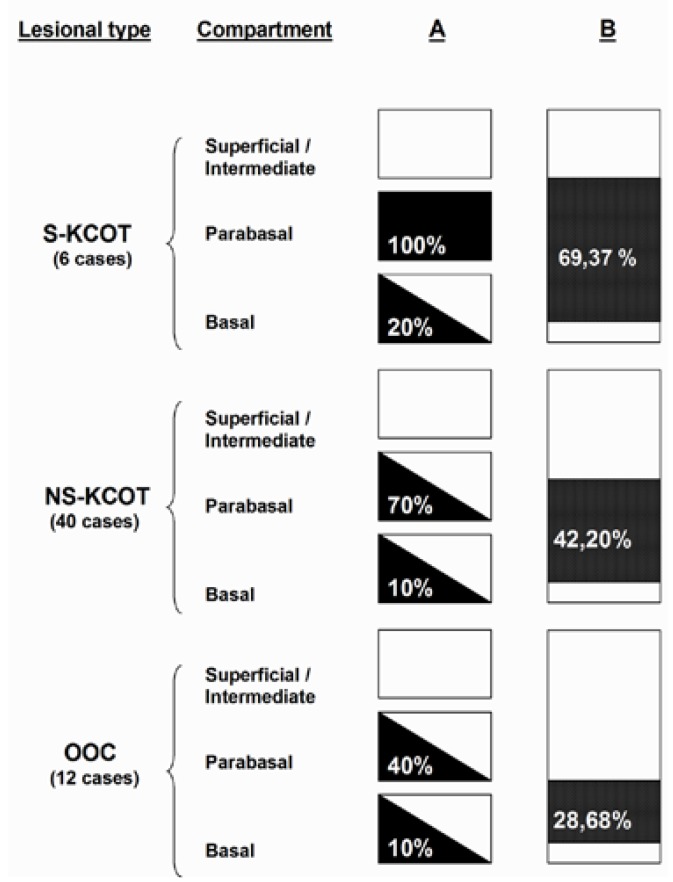
Schematic representation of CCD 1 expression by lesional types. In column A, epithelial thickness is divided into three compartments represented by boxes. CCD1 expression is represented by a 3-grade evaluation: the black shading represents that the expression was observed in virtually all the cells of compartment. The white shading represents that the expresión was almost completely negative in all compartment. The diagonal represents that the expression is partially positive in the compartment, the number indicate porcentage of CCD1+ cells. Column B represents total epithelial thickness, black shading indicates the porcentual thickness of epithelium CCD1+ and the level at which is located, the number indicate porcentage of thickness with CCD1+ immunostaining.

CCD1 expression was verified immunohistochemically in all cases (Figs. 1,2). The protein was expressed principally in the parabasal epithelial compartment, and (to a much reduced extent) in the basal compartment. CCD1 was not expressed in the intermediate/superficial level of the epithelium ( Table 2 and Fig. 3), summarized and schematized CCD1 results). The protein was expressed predominantly in the nucleus but was also present in the cytoplasm of the majority of cases (83.3%, 82.5%, and 75% of S-KCOT, NS-KCOT, and OOC cases, respectively), although nuclear staining was always more pronounced in intensity.

**Table 2:**
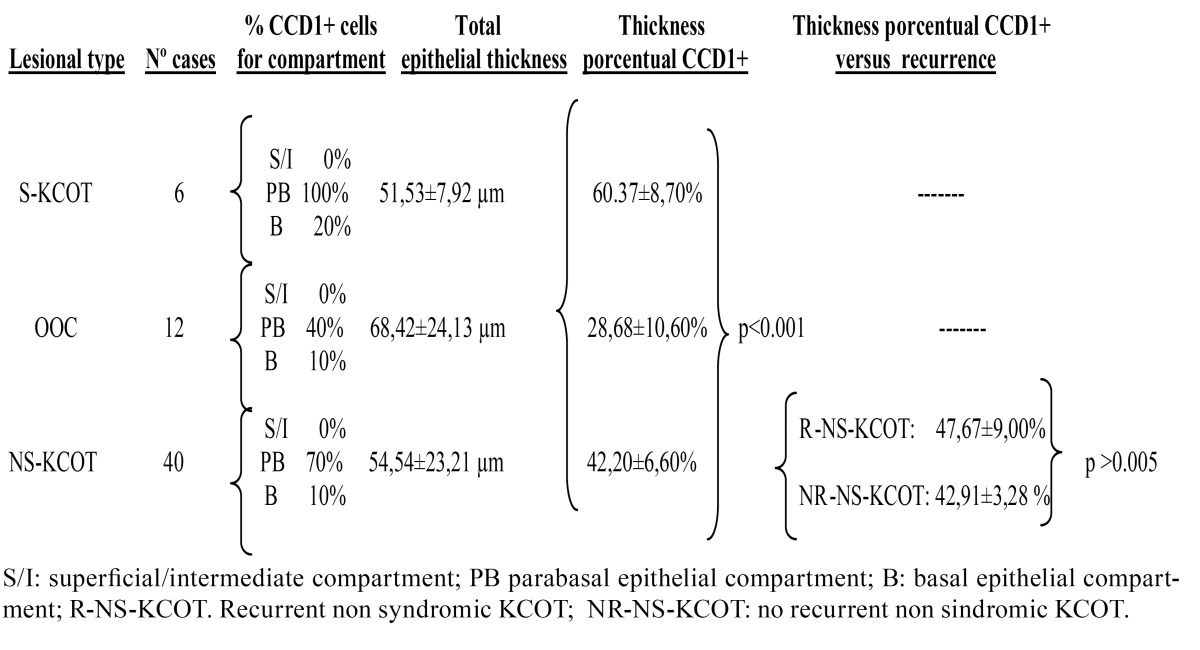
CCD1 immunohistochemical results by lesional types refers to percentage of positive cells in each epithelial compartment and porcentual thickness of epithelium with CCD1 immunostaining, as well as its relation with recurrence in NS-KCOT cases.

Virtually all parabasal cells of S-KCOT tissues were positive for CCD1, as were 70% and 40% of such cells of NS-KCOT and OOC tissues. The proportions of basal cells that immunostained for CCD1 were 20%, 10%, and 10% in S-KCOT, NS-KCOT, and OOC samples, respectively. S-KCOT epithelium stained to a depth of 60.37±8.70%, principally because the parabasal cell layer was thick, whereas the staining depths of NS-KCOT and OOC epithelia were 42.20±6.60% and 28.68±10.60% respectively, showing these three percentages of CCD1+ epithelial thickness, when compared one to one between them, statistically significant differences (*p*<0,001). The difference in the extent of staining between S-KCOT and NS-KCOT tissue was attributable principally to a difference in the thickness of the parabasal layer. However, the shallow staining of OOC tissue was explained not only by the presence of only a thin parabasal cell layer in such tissue but by the fact that the mean epithelial thickness of OOC tissue (68.42±24.13 µm) was greater than those of S-KCOT (51.53±7.92 µm) and NS-KCOT (54,54±23,21 µm) tissue. Finally, the epithelia of recurrent NS-KCOT (R-NS-KCOT) tissue (14 cases) stained to a mean depth of 47.67±9.00%, whereas that of non-recurrent tissue (NR-NS-KCOT) stained to a mean depth of 42,91±3,28; this difference was not statistically significant (*p*>0.005).

## Discussion

Keratin-producing odontogenic cysts are a heterogeneous group of lesions, that often show biological aggressivity with marked tendency to recur, and an important proliferative activity (1-3), which motivates that many forms are currently considered as true tumors (1,2,4).

Of the 58 lesions analyzed in the present study, 46 were histologically classified as KCOT using the WHO criteria (4), and, of these, 6 were considered to be S-KCOT because of the occurrence of other abnormalities (13). The remaining 12 cases were diagnosed as OOC based on histological criteria previously described (4-8). OOC is an orthokeratinized cystic variant that, unlike KCOT, has no syndromic association, does not recur, and does not form multiple lesions, being currently considered a separate entity (4-8). Our lesional distribution differs from that noted by others authors (14). In the cited work, S-KCOT accounted for 4-5% of all KCOT cases, although a systematic literature review found that the proportion was somewhat higher, being 6-11% (15). Likewise occurs in OOC, which is indicate represent approximately 10% of KCOT (7). Our data, in this sense, are likely influenced by to be cases treated in a single center, which acts as a referral hospital with a not population influence area.

The recurrence rates recorded in the present study are consistent with those reported in the literature. We found that OOC did not recur, as also noted by others (6,8), although a review of all published data noted isolated and rare recurrences of this condition (7). The NS-KCOT recurrence rate in our patients was 35%, very similar to that reported in other series with long follow-up intervals (16). Systematic reviews of NS-KCOT have considered that the average recurrence rate was 28% (7,15), but large among-series variations were evident, and recurrence is clearly influenced by the nature of the therapy performed (3). All S-KCOT patients studied by us experienced recurrences. Earlier reports found that the recurrence rates were 68–80% (16,17), although the fact that almost all patients have multiple lesions (17) and that the disease arises early in life suggest that the recurrence rate is influenced by the extent of monitoring (4). In our series, the follow-up duration was 112±76.1 months.

In the present study, we quantified CCD1 expression levels in a series of keratin-producing odontogenic cystic, many of which are today considered to be true tumors (1,2,4) whereas others are innocuous cystic lesions lacking the capacity to recur (5). Cell proliferative activity is a prognostic marker in many tumor types (18), and proliferation is closely associated with accelerated cellular growth kinetics. CCD1 is important in this context, being a nuclear protein essential for cell cycle progression through the G1 phase (11). In G1, CCD1 binds to and activates the cyclin-dependent kinase CDK 4/6, forming a complex that in turn phosphorylates the Rb protein, allowing progression to S phase and DNA replication (11,12).

Prior to the present study, CCD1 expression levels were analyzed principally in KCOT cases (14,19-23), and the results were sometimes contradictory, probably because of differences in methodology and the modes of data quantification used (14). Lo Muzio *et al*. (14) were the first to perform such an analysis; these authors found that CCD1 was expressed only in S-KCOT tissue, being completely absent from NS-KCOT samples. Thus, it appeared that CCD1 expression was specific to S-KCOT. These earlier data were negated in subsequent studies using different antibodies (19-21,24), including the EP12 antibody that we also employed. CCD1 was expressed by both S-KCOT and NS-KCOT tissue, but parabasal expression was more pronounced in S-KCOT samples. Thus, the two lesional types differ in CCD1 expression pattern.

In our study we found a gradual decrease in the extent of CCD1 expression (in terms of the depth of stained epithelium) from S-KCOT to NS-KCOT to OOC samples. This was associated with the fact that the thickness of the parabasal cell layer was greatest in S-KCOT tissue, less in NS-KCOT samples, and least in OOC tissue. Prior to our study, only Gani *et al*. (23) have been studied CCD1 expression simultaneously in OOC and KCOT samples. Their results are similar to those founds by us and these authors postulate that in KCOT occurs an asymmetric cell division at parabasal level, an attractive hypothesis that should be confirmed with other approaches in the future. Based on our results we postulate that a cellular dysregulation in terms of progression from the G1 to the S phase of the cell cycle was associated with variation in the extent of aggressiveness of the three types of keratin-producing odontogenic cysts. The CCD1 expression level of OOC tissue was rather low; only 28.68±10.60% of the total epithelial thickness was positive for the protein, thus lower than that noted in KCOT samples. This is in agreement with the observation that OOC cells have a lower proliferative activity than do KCOT cells, as has been shown using other methodologies (6,24).

In our observations, CCD1 expression was primarily nuclear, but weak cytoplasmic immunostaining was often evident, being similar in extent among the three lesional subtypes. Previous studies of keratocystic lesions (14,19-23) do not mention such findings, but similar nuclear and cytoplasmic staining has been noted in other lesions of different location (24), and it has been suggested that CCD1 may occur in the cytoplasm after the cell cycle is completed (23), or because genes involved in nuclear localization of CCD1 may have been inactivated (25).

In terms of recurrence of NS-KCOT, the CCD1 expression levels did not differ between R-NS-KCOT and NR-NS-KCOT samples. Only Kimi *et al*. (21) have examined CCD1 expression levels in recurrent and primary NS-KCOT samples, using a different quantification methodology, and also found that the CCD1 expression levels did not differ between the two types of lesions. Thus, CCD1 over expression does not seem to predict possible recurrence of NS-KCOT. Recurrence is probably influenced by factors unrelated to tumor biology, in this sense an adequate therapeutic management may effectively prevent recurrence of these non-syndromic lesions (4,14).

Finally, a recent genetic study (9) described three forms of KCOT. Type 1 disease, or S-KCOT, was associated with germline PTCH1 mutations; type 2 disease, or NS-KCOT, with loss of heterozygosity (LOH) of PTCH1; and type 3 disease (another form of NS-KCOT) with absence of mutation or LOH of PTCH1. Type 3 disease was in turn subdivided into subtypes 3A and 3B, based on the phenotypic expression patterns of cell-cycle-regulatory proteins (CCD1 and Bcl-2) in the basal and parabasal epithelial compartments. Although the cited study did not address OOC, which has also been associated with LOH of PTCH (10), it is interesting that the cited work highlighted the importance of differentiating CCD1 expression in the basal and parabasal compartments, as performed in our present work. Future genotypic analyses of larger series, coupled with analysis of CCD1 expression patterns, are needed to confirm the validity of the recent genotypic/phenotypic classification of keratin-producing odontogenic lesions.
